# Windblown Dust Deposition Forecasting and Spread of Contamination around Mine Tailings

**DOI:** 10.3390/atmos7020016

**Published:** 2016-01-28

**Authors:** Michael Stovern, Héctor Guzmán, Kyle P. Rine, Omar Felix, Matthew King, Wendell P. Ela, Eric A. Betterton, Avelino Eduardo Sáez

**Affiliations:** 1 Department of Atmospheric Sciences, University of Arizona, Tucson, AZ 85721, USA; 2 Department of Chemical and Environmental Engineering, University of Arizona, Tucson, AZ 85721, USA

**Keywords:** dust transport, mine tailings, forecasting, dust collection, arsenic and lead

## Abstract

Wind erosion, transport and deposition of windblown dust from anthropogenic sources, such as mine tailings impoundments, can have significant effects on the surrounding environment. The lack of vegetation and the vertical protrusion of the mine tailings above the neighboring terrain make the tailings susceptible to wind erosion. Modeling the erosion, transport and deposition of particulate matter from mine tailings is a challenge for many reasons, including heterogeneity of the soil surface, vegetative canopy coverage, dynamic meteorological conditions and topographic influences. In this work, a previously developed Deposition Forecasting Model (DFM) that is specifically designed to model the transport of particulate matter from mine tailings impoundments is verified using dust collection and topsoil measurements. The DFM is initialized using data from an operational Weather Research and Forecasting (WRF) model. The forecast deposition patterns are compared to dust collected by inverted-disc samplers and determined through gravimetric, chemical composition and lead isotopic analysis. The DFM is capable of predicting dust deposition patterns from the tailings impoundment to the surrounding area. The methodology and approach employed in this work can be generalized to other contaminated sites from which dust transport to the local environment can be assessed as a potential route for human exposure.

## 1. Introduction

Wind erosion, transport and deposition of particulate matter from contaminated sites may have significant effects on the surrounding environment, especially in arid and semi-arid regions, which are especially susceptible to erosion because of the dry climate and lack of vegetation. Wind erosion occurs on a variety of spatial scales from very large dust storms that can travel thousands of kilometers in the atmosphere [1] to small local sources whose impact is regionally confined. Some of the human health concerns associated with elevated concentrations of particulate matter include fungi and bacteria transport [2] and respiratory stress and cardiovascular disease [3]. Local sources of windblown dust such as dry lake beds, plowed fields and mine tailings can regularly produce windblown particulate matter [4,5]. In semi-arid mining regions, such as the US Southwest, mine tailings impoundments can be a significant anthropogenic local source of windblown particulate matter [4].

Modeling wind erosion, transport and deposition of particulate matter from mine tailings impoundments on scales of a few kilometers is challenging. Typical regulatory models, such as CALPUFF and AERMOD, have difficulty simulating aerosol transport in topographically complex regions on such small scales. Recent advancements in computational capabilities have made Computational Fluid Dynamic (CFD) modeling a viable approach for simulating the erosion and transport of aerosols but minimal research has been done on the modeling of wind erosion and aerosol transport from mine tailings impoundments. Previous studies that have investigated wind erosion of tailings impoundments through the use of CFD modeling [6–9] focused on the erosion process and not the transport and deposition of the windblown particulate matter. In a previous work [10], we utilized a CFD model to simulate wind erosion of a tailings impoundment in a topographically complex region, in order to understand the mechanisms that influence deposition.

The prediction of transport and deposition of windblown dust from mine tailings impoundments is vital in determining the exposure risks in neighboring communities. In a previous work [11] we developed a windblown dust Deposition Forecasting Model (DFM) that was designed to be used in conjunction with operational weather models to forecast deposition of windblown particulate matter for the Iron King Mine (IK) and Humboldt smelter tailings impoundments in Dewey-Humboldt, Arizona. The DFM is a hybrid model that uses both empirical relations derived from direct observations and physical model simulations of aerosol trajectories. The IK and Humboldt smelter tailings impoundments are part of a US Environmental Protection Agency Superfund site that has elevated concentrations of toxic species such as lead and arsenic [12].

In this work, we assess predictions of the DFM by validating the deposition forecasts against a variety of field measurements at the mine site and surrounding areas, including: (i) spatially distributed measurements of dust deposition; (ii) metals composition; and (iii) lead isotope analysis. The spatially distributed measurements of dust deposition were collected using inverted-disc samplers during two month-long field sampling campaigns in April and June 2014. The sampling periods were selected to coincide with spring and early summer, which represent the windy season in southern Arizona. Dust collected during the sampling campaigns was analyzed for total weight, chemical composition and lead isotopes and directly compared to forecasted deposition patterns generated by the DFM. We hypothesize that forecast spatial deposition patterns generated by the DFM will agree with arsenic and lead tracers captured by the inverted-disc samplers, which will help in establishing routes of contaminant exposure in local communities. In addition, we investigate how topsoil measurements of arsenic and lead performed in this work based on techniques developed in a previous work [13] correlate with the dust sampler contaminant concentrations and isotopic signatures, which serves to quantify the spread of contamination from the mine tailings site.

## 2. Methodology

### 2.1. Site Description

**Figure 1:**
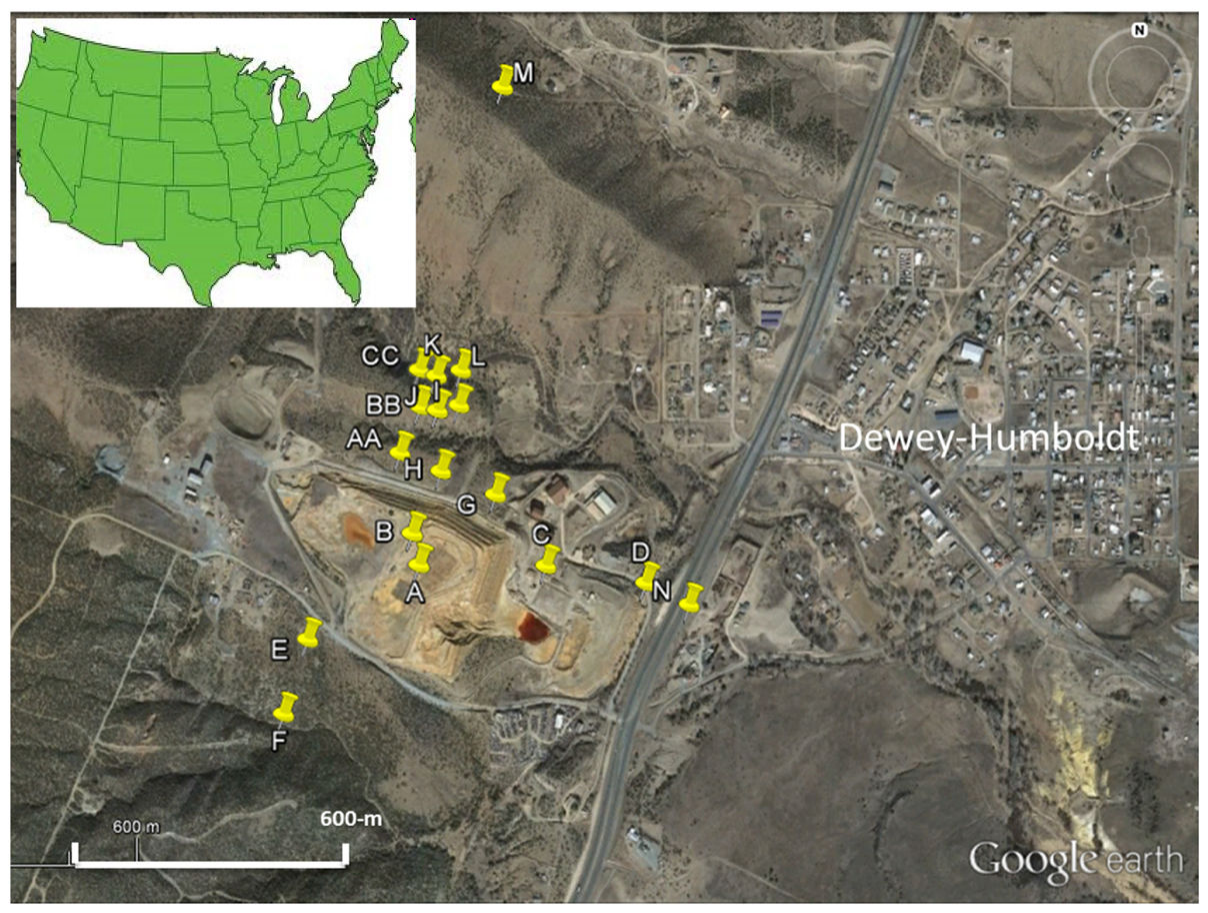
Google Earth visible satellite image of the Iron King Mine tailings impoundment and the town of Dewey-Humboldt (March 2014). The locations of the inverted-disc samplers are denoted by yellow pins (image from Google Earth).

The Iron King Mine Tailings and Humboldt Smelter Superfund area is located in central Arizona in the vicinity of Dewey-Humboldt (Figure 1). The smelter produced lead, gold, silver, zinc and copper in the period 1906–1969. The area was classified as a Superfund site by the US EPA in 2008 [12] after it was discovered that it is contaminated with lead and arsenic.

The region is classified as semiarid with an annual rainfall of about 480 mm. The vegetation is Pinyon Juniper woodlands with limited desert grasses and other bushes [14]. Most of the land adjacent to the northern, western and southern edges of the tailings and mine operations property is publicly owned state trust and grazing land. Arizona State Highway 69 separates the mine tailings area from the town of Dewey-Humboldt, which has an elevation of 1396 m.

The tailings impoundment consists of two areas: the main tailings impoundment with a total aerial extent of 96,000 m^2^ and the lower tailings region located directly adjacent to the main tailings impoundment with a total aerial extent of 84,000 m^2^. The tailings impoundment is devoid of vegetation except where a phytostabilization project is attempting to reestablish native vegetation. The revegetation project has been in progress since May 2010 and is confined to an area of 7200 m^2^ on top of the tailings [15]. The surface of the impoundment is made up of 34.7% sand, 44.8% silt, and 20.4% clay and has patches of reddish coloration attributed to iron minerals, mostly ferrihydrite [15]. This type of tailings material composition is highly skewed towards the fine particle size range with 65.2% being silt size (<50 μm). The tailings are typically covered by crusted soil with patchy efflorescence that usually forms following rain events. The crust can be broken up and results in very fine powdery material that is easily eroded by the wind. The average arsenic and lead concentrations measured in bulk samples of the mine tailing material are about 0.12% and 0.10% by mass, respectively.

A suite of meteorological and dust monitoring instruments was installed on the tailings impoundment in 2009. The setup consists of two eddy flux towers, equipped with six DUSTTRAK dust monitors for PM_27_ (particulate matter with an aerodynamic diameter less than 27 μm,) anemometers, wind vanes, thermometers and hygrometers. In addition, a Micro-Orifice Uniform-Deposit Impactor (MOUDI) was deployed to measure size fractionated distribution of aeolian dust from April 2013 to January 2014. Four month-long MOUDI samples were collected during this period. The MOUDI (MSP Corp) has eleven stages that collect particles with aerodynamic cut point diameters of: 18-μm, 9.9-μm, 6.2-μm, 3.1-μm, 1.8-μm, 1-μm, 0.55-μm, 0.32-μm, 0.18-μm, 0.1-μm, 0.054-μm and ultrafine particles collected on an “after filter”. The MOUDI was located along the northern edge of the tailings impoundment with the inlet located 1 m above ground level. Observations collected by the DUSTTRAK and the MOUDI were used to generate empirical relations between dust concentration (discriminated by particle size), wind speed at 10-m height and relative humidity. These correlations were employed in the calculation of dust fluxes in the DFM [11]. In addition, the meteorological observations are used to determine systematic biases between observed conditions and operational WRF forecasts.

### 2.2. Deposition Forecasting Model Description

The DFM predicts the deposition of PM_27_ generated by windblown dust from the Iron King tailings impoundment. This size range has been chosen since it corresponds with the measurements made by the DUSTTRAK dust monitors. The hybrid model utilizes both empirically derived relations and particle transport simulations. Details of model development and validation are presented elsewhere [11].

The nearly two years (2012–2013) of meteorological and dust monitoring data collected from the eddy flux towers were used to derive empirical relationships between meteorological conditions and windblown dust generation. These empirical relations include the effects of wind speed and relative humidity on airborne dust, particle size fractionation and the vertical profile of dust concentration measured on the tailings and wind speed effects on wind direction. Here, we will summarize the empirical correlations presented in our previous work [11]. We correlated dust concentrations in the atmosphere over the tailings surface to obtain

(1) $$C_{\mathrm{dust}}=A_{1}A_{2}U^{B}$$

where C_dust_ is the mass concentration (μg/m^3^) of dust in the atmosphere at a specific height and for a specific particle size range, U is the 10-m height wind speed, A_1_ and B are constants that are fitted to measurements for each specific height and particle size range (see [11] for values and details), and A_2_ is a function of atmospheric relative humidity, empirically found to be:

(2) $$A_{2}=\left\{ \begin{array}{c} 1\;\mathrm{for}\;RH\leq 25\%\\ (50-(RH-25))/50\;\mathrm{for}\;25\%<RH\leq 75\%\\ 0\;\mathrm{for}\;RH>75\% \end{array}\right.$$

where RH is relative humidity (%). Concentrations, wind speeds and relative humidities in these empirical equations correspond to 1-h averages.

The DFM forecasts a dust emission flux from the tailings as proportional to the integral of the concentration given by Equation (1) over height. The flux is assumed to be uniform over the whole surface area of tailings (95,000 m^2^).

The DFM forecasts deposition in three particle size ranges. The size ranges are defined by particles that have aerodynamic diameters (D_p_) that fall within: 27 μm > D_p_ > 18 μm (coarse), 18 μm > D_p_ > 3.1 μm (medium), and D_p_ < 3.1 μm (fine). The size ranges were determined by MOUDI and DUSTTRAK observation capabilities: The coarse range represents the difference between DUSTTRAK and total MOUDI mass measured in a given event (that is, for the same amount of air treated). The medium and fine ranges were obtained by adding the mass of collected sample in corresponding stages in the MOUDI. The cut point (3.1 μm) was selected based on the fact that, on average, total mass collected by the MOUDI above and below that particle size was approximately the same. In addition, the same size ranges were used in deriving the empirical relationships between meteorological conditions and wind erosion flux within the DFM model [11].

The DFM also includes the effect that topography has on aerosol deposition. Stovern *et al.* [10] simulated windblown transport of fugitive aerosols from the Iron King Mine tailings using computational fluid dynamics. They found that windblown dust preferentially deposits in regions of topographic upslope relative to the mean flow. Due to the complex topography of the site, these effects are included in the model.

The DFM results for each forecast period (see Section 3) are produced for three particle size ranges and are modelled on a spatial grid that is approximately 25 km^2^ in area with 10.3 m spatial resolution. These simulations include the effects that a convectively turbulent boundary layer has on particle trajectories. Simulation area includes all of the Iron King tailings and most of the adjacent town of Dewey-Humboldt. The model is initialized using the 48-h forecasts from an operational version of the WRF model produced by the department of Atmospheric Sciences at the University of Arizona.

### 2.3. WRF Model Forecasts

The WRF model is configured with two nested grids. The inner grid covers the entire state of Arizona, portions of Southeastern California, Western New Mexico and Northern Mexico. The outer grid covers from Northern Nevada to the tip of the Baja California peninsula, and from Western Texas to portions of the Eastern Pacific Ocean. The inner and outer domains have horizontal resolutions of 1.8-km and 5.4-km, respectively. Model forecasts are produced daily at 12Z and 6Z, using both Global Forecast System (GFS) and North American Mesoscale Forecast System (NAM) initializations. Each forecast run is 48 h long at 1-h intervals. The DFM was initialized using the WRF forecast conditions predicted 28–39 h in advance.

We used the daily 12Z GFS WRF runs during the periods 21 April to 22 May and 11 June to 9 July 2014 to tabulate the horizontal components of the 10-m height winds, 2-m specific humidity, the 2-m height temperature and the surface pressure for each hour between 9 A.M. and 9 P.M. We then calculated the wind speed and wind direction and relative humidity for each hourly interval by averaging the five nearest-neighbor WRF grid points at the tailings location, which was used to initialize the deposition forecasting model.

WRF model forecasts and observed meteorological conditions on the tailings were compared to test for systematic biases over a period of 163 days (June 2012 to August 2013). This period coincides with the same observing period used to determine the empirical relations used in the creation of the deposition forecasting model [11]. As described later, forecast and observed wind speed and relative humidity were corrected for systematic bias. Results will be summarized in Section 3.1. To calculate directional bias we first calculated the residual for each model and observation pair which fall within the range of −180 to +180 degrees. A positive residual indicates counter-clockwise rotation between the WRF model and observed wind direction and *vice versa*. A perfectly nonbiased data set would produce an average residual of zero.

### 2.4. Dust Deposition Sampling

Inverted-disc samplers have been used in dust deposition experiments, including aeolian deposition near an eroding source field [16] and dry deposition of polychlorinated organics [17]. The collection efficiencies of inverted-disc samplers have been studied extensively [18–22] and are dependent on wind speed and particle diameter. However, there appears to be general consensus that the collection efficiency falls within the range of 5%–40% for all grain sizes [21]. For this study, collection efficiency is unimportant because we base our comparisons on relative deposition amounts (Section 3.3.2). Hall and Waters [19] showed that an inverted-disc sampler has a significantly higher blowout wind speed than both the flat disc and the British Standard deposit gauge. This reduces resuspension and loss of particulate matter.

The plastic discs (Frisbees) were purchased from discountmugs.com. They have a diameter of 233 mm and a depth of 25 mm. The discs were glued to the lids of high density polyethylene 500-mL sample bottles (Thermo Scientific) using Loctite plastic bonder. An 8.5 mm hole was drilled through the disc into the bottle. The samplers were mounted on iron stakes and placed 1 m above the ground.

A set of 20 inverted-disc samplers was placed around the Iron King tailings impoundment and surrounding area. The disc samplers were placed along three transects to measure deposition in the northward, eastward and southward directions, starting from the main tailings and extending up to 1 km away. A majority of the samplers were located north of the main tailings, along the dominant wind direction (Figure 1). There is significant topographic variation north of the tailings, which is used for testing the effect that topography has on dust deposition.

### 2.5. Soil Sampling

Soil samples were taken during a single sampling campaign in 2014 at different distances from the mine tailings to assess the extent of contamination. A total of nine soil samples were collected with the first sampling point located on the mine tailings and eight more sampling points in a straight line NE transect, which corresponds to the prevailing wind direction. Samples at different depths were taken in order to obtain a vertical profile of the contamination at the following depth intervals: 0–3 mm, 3–6 mm, 6–9 mm and finally 100 mm. Details of the sampling technique are provided elsewhere [13]. Final samples at each site and depth were a composite of three different samples. The composite samples were dried for 10 h at a temperature of 110°C and then sieved through a 0.84 mm sieve to discard coarser fractions. Distances from the tailings to the sampling points along the NE transect were 13 m, 70 m, 130 m, 355 m, 1100 m, 1128 m, 1150 m and 5032 m.

### 2.6. Sample Analysis

In the field, deionized water was used to flush all the dust captured on the inverted-disc samplers into the attached 500 mL bottle. The bottles were unscrewed and capped for transportation to the laboratory where each sample was partially dried in the 500 mL bottle in an oven (60°C) and then transferred into a pre-weighed 50 mL glass vial. The vial samples were then completely dried and sieved to remove particles >500 μm. The samples were then weighed using a Mettler AE100 balance (±0.1 mg). The sample masses were normalized to the area of the disc, which yields the mass deposition per unit area for each sample location. The handling of MOUDI samples has been described in detail elsewhere [4].

Both inverted-disc and soil samples were prepared for metal and lead isotopes analysis by extraction with 15 mL of *aqua regia* (1.03 M HNO_3_/2.23 M HCl, trace-metal grade) with sonication at 80°C for 60 min. Aliquots of 1.2 mL of solution were extracted and diluted to 4 mL with deionized water before the analysis. Due to the relatively low concentrations of lead in some of the samples, the lead isotope analysis samples were concentrated on a hot plate [13]. An ICP-MS (Agilent 7700X with an Octopole Reaction System) was used to analyze for metal concentrations and lead isotopic composition. MiliQ water, 0.669 HCl (Fisher, trace-metal grade) and 0.309 M HNO_3_ (EMD, Omnitrace) were used to create the certified calibration standards from Accustandard. In addition to each sample, the National Institute of Standards and Technology (NIST) standard reference material (SRM 1643e trace elements in water) was also analyzed. We used the same operating condition for the analysis of both elemental concentrations and the lead isotopic ratios. NIST SRM 981 (Lead isotopic standard) was used for validation and calibration and the analytical precision of lead isotopic ratios was under 0.5% [13].

## 3. Results

### 3.1. WRF Model Verification

**Figure 2:**
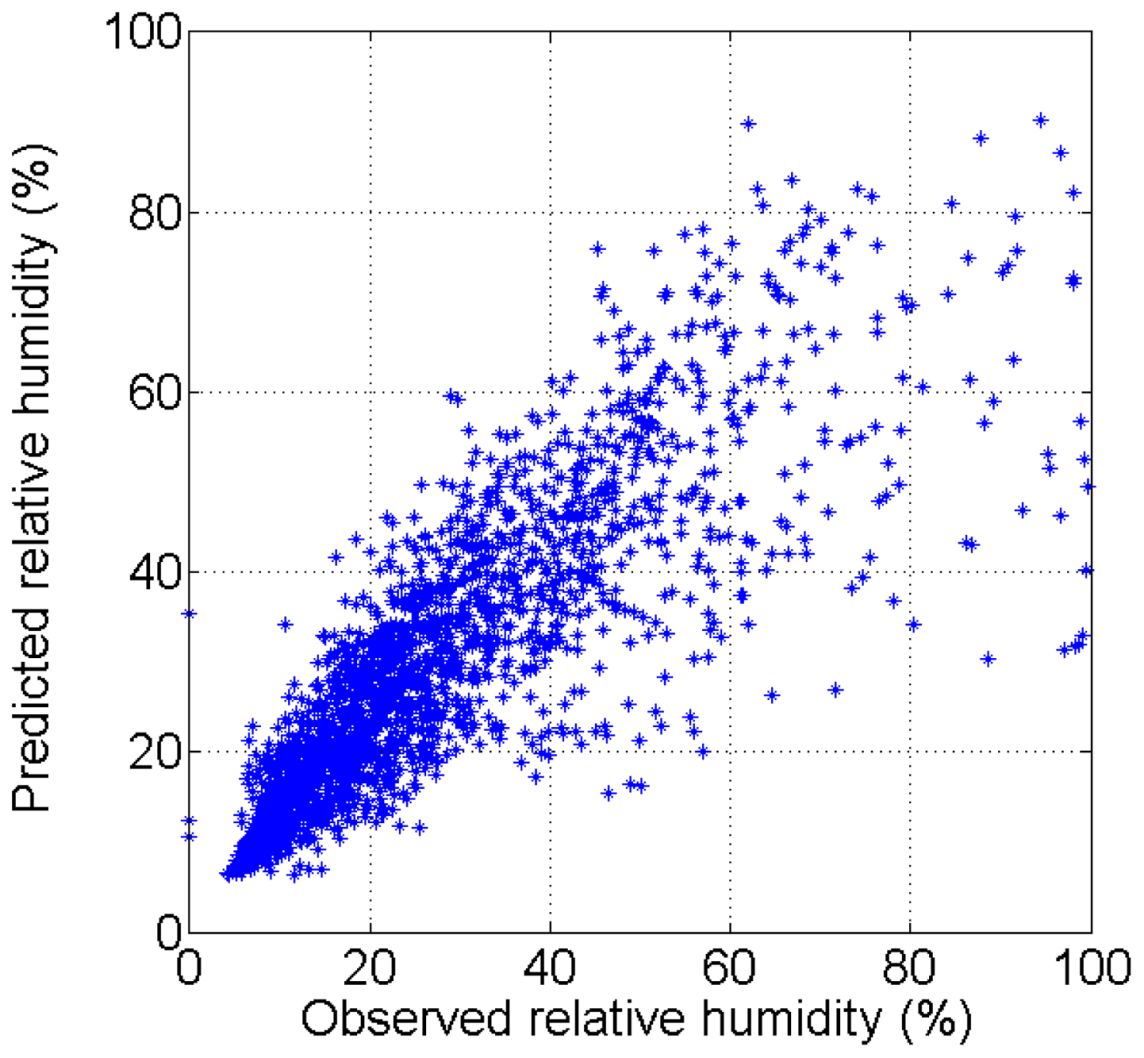
Comparison of hourly averaged observed relative humidity and WRF model forecast predictions for the period of 29 May 2012 to 4 August 2013. A linear fit yields a slope of 0.97 with R^2^ = 0.59.

Biases in the 24-h WRF forecast of wind speed, wind direction and relative humidity are determined by direct comparison to *in situ* observations. The forecast of relative humidity showed good agreement with the observations (Figure 2). The results show that the WRF model slightly under predicts the observed relative humidity, which may be caused by precipitation events that the WRF model failed to forecast since potential dust mitigation due to remnant soil moisture from past rain events are not included in the model. To adjust for this slight bias in our calculations, the model-predicted relative humidity is multiplied by a correction factor of 1/0.97, product of a least-squared error fit of the parity plot in Figure 2.

**Figure 3:**
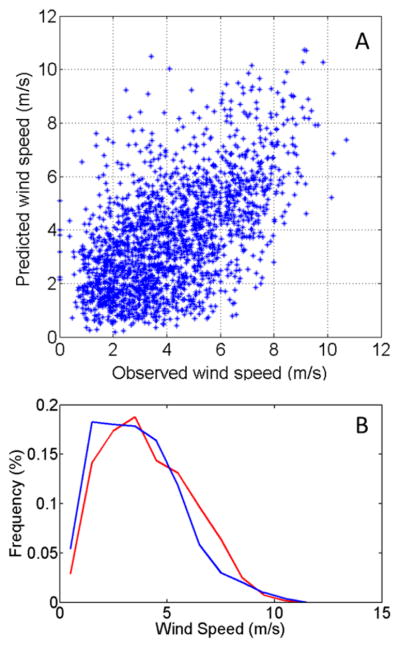
(**A**) Direct comparison of hourly averaged 10-m height observed wind speed and WRF model predictions for the period of 29 May 2012 to 4 August 2013. A linear fit yields a slope of 0.84 with R^2^ = 0.23; (**B**) Frequency distribution of hourly averaged 10-m height observed wind speed and WRF model predictions for the same period.

The forecast and observed hourly averaged wind speeds are also correlated but with a high degree of scatter (Figure 3A). The results show that the WRF forecast winds are systematically lower than those observed (slope < 1). The low R^2^ value is indicative of the difficulties when comparing model forecasts using 1.8-km grid spacing to point observations where boundary layer mechanics and surface roughness play an important role. The frequency distribution of the model forecast and the observed wind speeds, Figure 3B, shows that over multiple hourly periods the distributions are very similar. The model-predicted wind speed is corrected in our calculations by multiplying by 1/0.84, which is the fitted slope of the parity plot (Figure 3A).

**Figure 4:**
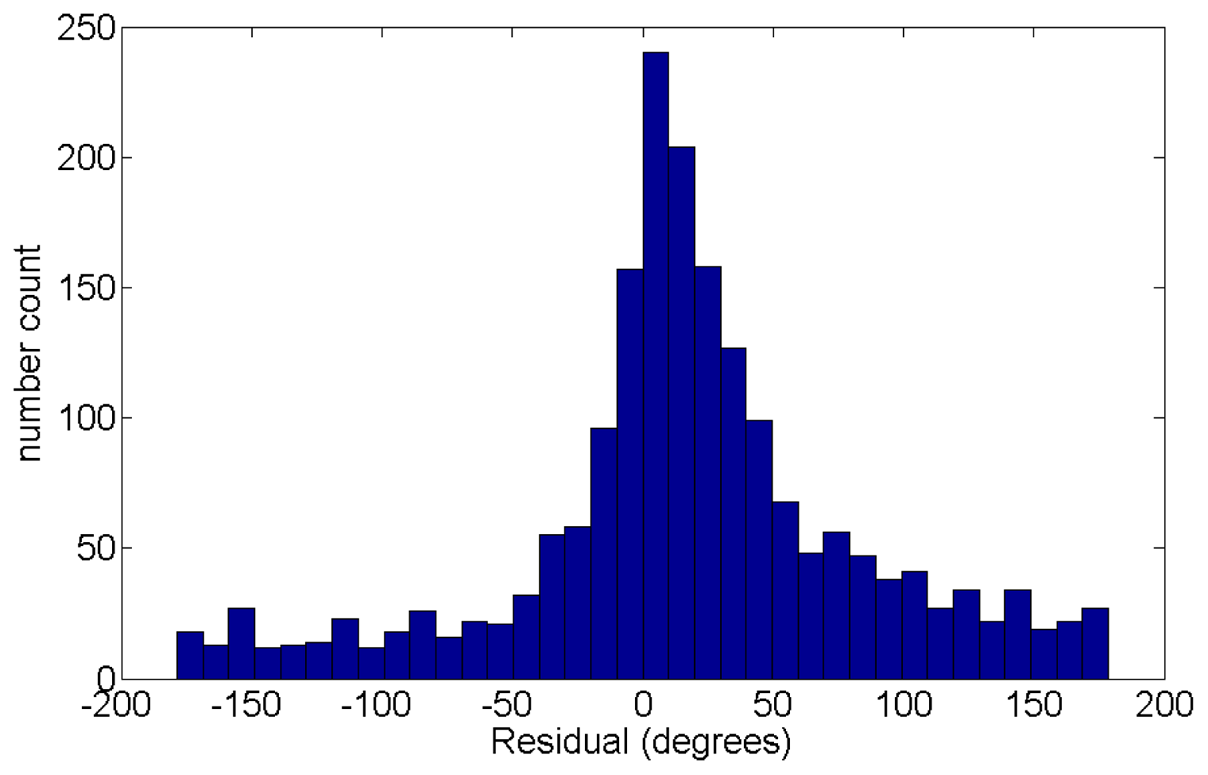
Histogram of residuals of measured wind direction (1-h averages) and WRF model predictions for the period of 29 May 2012 to 4 August 2013. The displaced maximum is at +14.7°, which indicates a counter clockwise bias in the model results.

The histogram of the hourly wind direction residuals is shown in Figure 4. The histogram was generated using 10 degree bins from −180° to 180°. The histogram is shifted to the right of zero, which means the model-predicted wind direction is systematically biased counter clockwise from the observed. By adding 14.7°of clockwise rotation to the forecast wind direction (first moment of the histogram) we account for the apparent bias between model forecast and observed conditions.

### 3.2. Deposition Model Predictions

**Figure 5:**
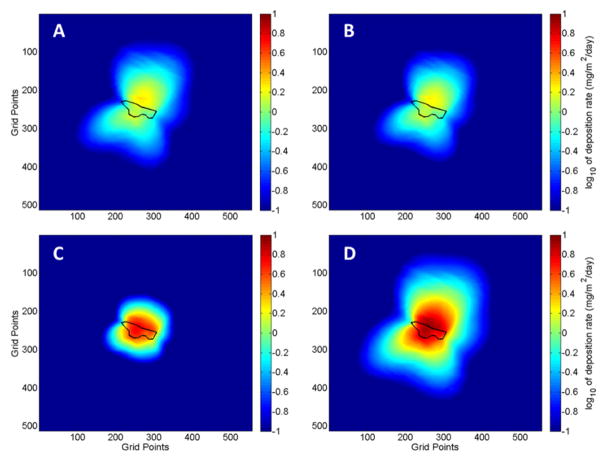
Maps of predicted dust deposition around the IK mine tailings for three size fractions and PM_27_ predicted by the DFM for the forecast period 21 April to 22 May 2014. Particle size ranges: (**A**) PM_3.1_; (**B**) PM_3.1_–PM_18_; (**C**) PM_18_–PM_27_; (**D**) PM_27_. The tailings impoundment is outlined in black. The grid points are spaced by 10.3 m and the domain has a total horizontal extent of 34.47639° to 34.52380° latitude and −112.27639° to −112.22491° longitude. Calculated dust deposition corresponds to dust emitted from the mine tailings.

The DFM simulations were initialized using the corrected WRF model forecasts. Figure 5 shows deposition patterns predicted by the DFM for the fine (PM_3.1_), medium (PM_18_–PM_3.1_), coarse (PM_27_–PM_18_) and total suspended fine particulate (PM_27_) for the period 21 April to 22 May 2014. A majority of the deposition occurs in the northward direction, which is the prevailing wind direction for the period. However, there is significant predicted deposition in the southeastward and southwestward directions, which was a result of several synoptic scale troughs that shifted the daytime wind direction. One of these troughs was accompanied by precipitation on 27 April 2014. The WRF model predicted the strong wind speeds associated with the trough but failed to accurately forecast the precipitation that was observed at the site. The increased soil moisture caused by the precipitation greatly reduces windblown erosion. Hence, the deposition in the southwesterly direction is thought to be significantly overestimated.

The maximum coarse particle deposition was mainly constrained to the immediate vicinity of the tailings due to their large size and fast settling times, which do not allow for long distance transport. The fine particles have a much smaller terminal velocity and are transported much further downwind. Particles of 3-μm size have a maximum deposition location that is about three hundred meters from the tailings in the northward direction. Smaller particles may travel longer distances. There are slight variations in the deposition patterns caused by the impact of topography of the surrounding region.

**Figure 6:**
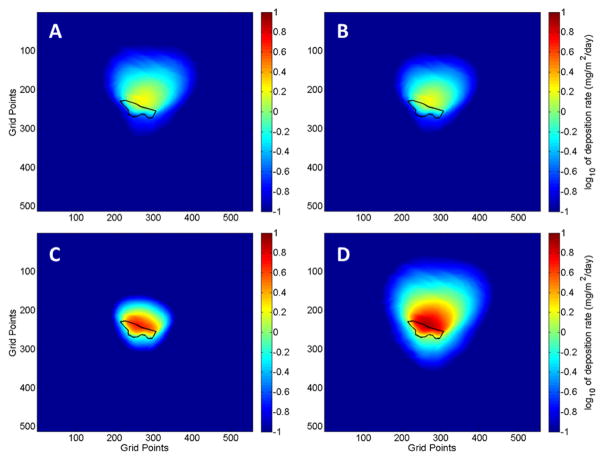
Maps of predicted dust deposition around the IK mine tailings for three size fractions and PM_27_ predicted by the DFM for the forecast period 11 June to 9 July 2014. Particle size ranges: (**A**) PM_3.1_; (**B**) PM_3.1_–PM_18_; (**C**) PM_18_–PM_27_; (**D**) PM_27_. The tailings impoundment is outline in black. The grid points are spaced by 10.3 m and the domain has a total horizontal extent of 34.47639° to 34.52380° latitude and −112.27639° to −112.22491° longitude. Calculated dust deposition corresponds to dust emitted from the mine tailings.

The resulting DFM simulations for the sampling period 11 June to 9 July 2014 can be seen in Figure 6. Compared to the May sampling period, the forecast weather conditions at the site were consistent with predominantly southerly winds that caused almost all the deposition to be in the northward direction.

### 3.3. Inverted Disc and Soil Sampling Results

#### 3.3.1. Mass Analysis

Inverted-disc sampling occurred during the sampling periods 21 April to 22 May 2014; and 11 June to 9 July 2014. Table 1 shows the deposition fluxes (mass deposited per unit time and sampler surface area) for each disc sampler during both periods and the corresponding model predicted PM_27_ deposition. It is important to point out that the model developed here predicts deposition of dust generated on the mine tailings and not total deposition, since the region has multiple potential dust sources. It is clear from the data in Table 1 that the model under predicts total deposition, as expected.

The highest deposition fluxes were measured at location N (Figure 1) for the May sampling period and location B for the June sampling period. Sample N is located adjacent to the highway while sample B is located on the main tailings impoundment. Because roadways are well documented as production source of atmospheric aerosols, it follows that we would expect larger amounts of deposition to be captured with the N sampler.

The observed deposition flux for the two sampling periods is within an order of magnitude of the forecast deposition fluxes. The peak forecast deposition flux is approximately half of that measured using the samplers. However, the model forecast deposition fluxes drops dramatically for samplers located far from the tailings source (*i.e.*, samplers F, M and N) while the total mass of dust measured by the samplers remains similar no matter their location. It is important to consider the fact that wind erosion occurs from a variety of sources within the region and each deposition sampler is collecting dust from all of them not just aerosols resulting from the tailings, although the exposed, elevated tailings are thought to be an important contributor.

As mentioned, the deposition forecasting model only simulates the transport of windblown particulate matter from the tailing impoundment and for particles with an aerodynamic diameter ≤27 μm. Comparing forecasts of PM_27_ to bulk deposition samples has inherent errors caused by the potential of the samplers to collect larger particles and skewing the mass fluxes. In order to minimize these issues certain steps were taken to reduce the impact of large particles. The inverted-disc samplers were shown in wind tunnel tests to have the highest collection efficiency, up to 60%, for particles with diameter 10 to 31 μm. Their collection efficiency significantly drops for particles up to 89-μm [21]. The samplers were placed at 1-m height to minimize the capture of large saltating particles. Additionally, mass fractions of dust measured by the MOUDI at 1-m height [11] were 30.1%, 30.0%, and 39.9% for the >18-μm, 18 to 3.1-μm and <3.1-μm size fractions. The large percentage of mass observed in the smaller size fractions gives us confidence that the inverted-disc samplers are not significantly affected by larger particles and their results are reasonable approximations of PM_27_ aerosol deposition.

Additional errors in the forecast depositions can arise from the DFM dependency on the WRF wind speed forecast. The WRF model provides us with the highest resolution weather forecasts of the region but it is still susceptible to errors including those experienced during short term high wind events. The compounding factors of multiple dust sources, DFM forecast of PM_27_ and errors in the WRF forecast makes the under estimated DFM deposition fluxes measured by the inverted-disc samplers reasonable, Table 1. Through the use of elemental analysis we can partition the captured dust and determine the relative influence the tailings have on each sampler and the relative spatial distributions.

#### 3.3.2. Lead and Arsenic Analysis

**Figure 7:**
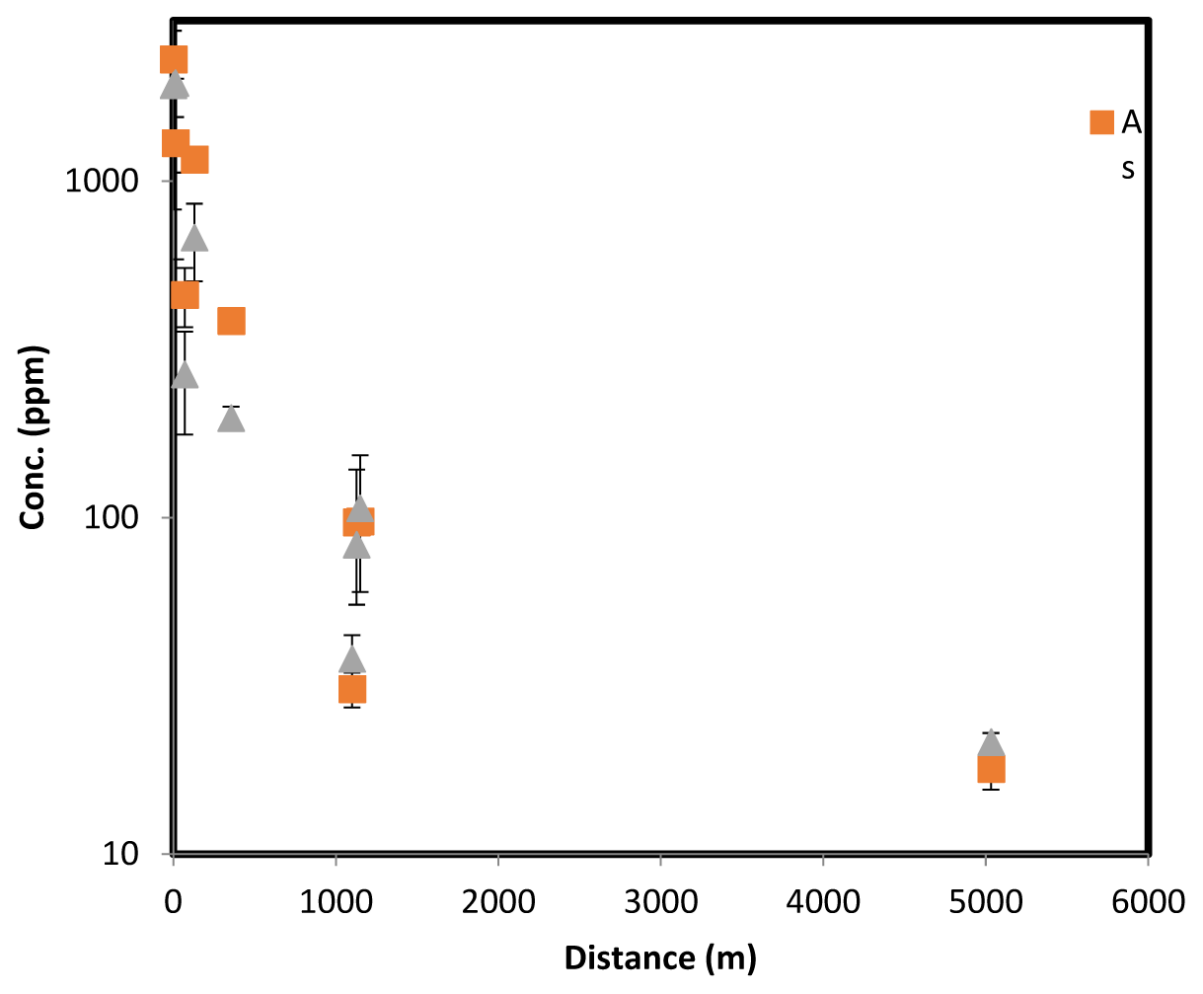
Arsenic and lead concentrations in topsoil (0–3 mm depth) samples at different distances from the mine tailings following a NE transect. Error bars show standard deviation of triplicate samples at the same location.

The tailings and surrounding soil have significantly elevated arsenic and lead concentrations when compared to the natural surroundings [14]. This is illustrated by the results in Figure 7, where topsoil concentrations of lead and arsenic are shown as a function of distance from the tailings along a NE transect. Significant arsenic and lead contamination extend at least to 1 km from the tailings, while the 5 km site exhibits relatively low concentrations, which could correspond to background levels in the region (around 10 ppm for both arsenic and lead).

**Figure 8:**
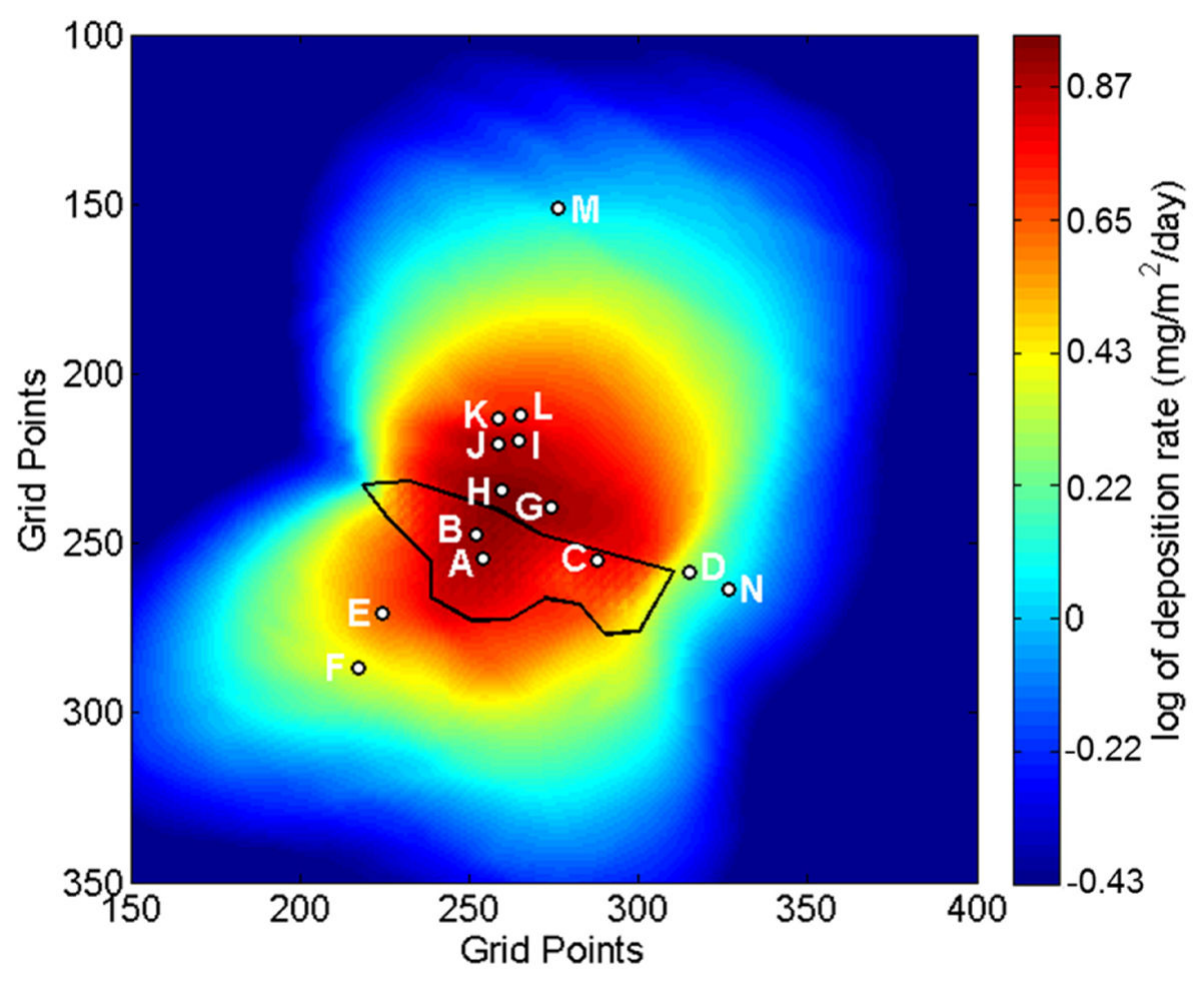
Map of PM_27_ deposition predicted by the DFM for the forecast period 21 April to 22 May 2014. The color scale represents the natural log of the deposition flux. The tailings impoundment is outlined in black and the locations of the inverted-disc samplers are indicated. The grid points are spaced by 10.3-m and the domain has a total horizontal extent of 34.49142° to 34.51452° latitude and −112.26247° to −112.23939° longitude.

**Figure 9:**
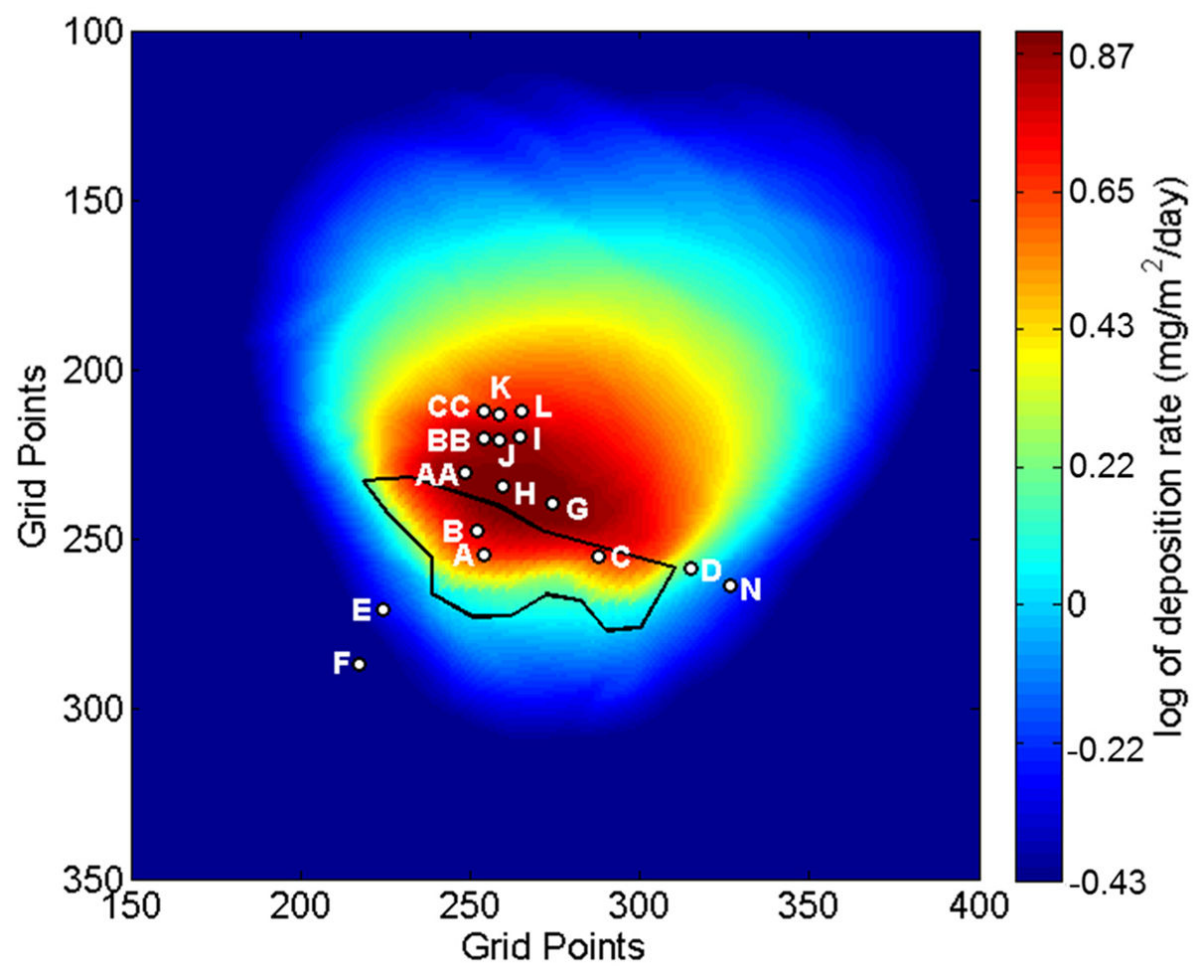
Map of PM_27_ deposition predicted by the DFM for the forecast period 11 June to 9 July 2014. The color scale represents the natural log of deposition. The tailings impoundment is outlined in black and the location and sample label of the inverted-disc samplers are indicated. The grid points are spaced by 10.3-m and the domain has a total horizontal extent of 34.49142° to 34.51452° latitude and −112.26247° to −112.23939° longitude.

The arsenic and lead concentrations measured in inverted-disc samplers are presented in Table 2. Figures 8 and 9 show the DFM predictions of average deposition fluxes for the May and June sample periods. A way to assess the results is to compare transects of relative observed concentrations of As and Pb with forecast deposition of PM_27_. Three transects are evaluated in the southwestward, eastward and northward directions. The relative Pb and As concentration transects are calculated by normalizing each sampler to the average concentration measured by inverted-disc samplers A and B located on the tailings pile. Equation (1) shows the normalization process for a sample concentration (C_sample_) where C_A_ and C_B_ are the concentrations measured at samplers A and B, respectively. Transects of the DFM PM_27_ are normalized by the forecast deposition at the location of sampler B (34.50087° latitude and −112.25305° longitude). All distances of the sample locations are calculated from sample point B.

(3) $$C_{\mathrm{sample}\;\mathrm{norm}}=\frac{C_{\mathrm{sample}}}{(C_{A}+C_{b})/2}$$

The southward transect is generated using the normalized samples A and B, E (366-m downwind) and F (538-m downwind). The eastward transect was generated using the normalized samples A and B, C (377-m downwind), D (657-m downwind) and N (786-m downwind). For the June sample period, the northward transect was generated by first averaging the sample concentrations collected at different downwind ranges, which included the AA samples (~205-m downwind), I, J, and BB samples (~300-m downwind) and the K, L and CC samples (~379-m downwind). The sample averaged concentrations at tailings, 205-m, 300-m and 379-m downwind were then normalized and used to generate the northward transect.

For the May sampling period we first averaged the sample concentrations of I and J (~300-m downwind) and K and L (~379-m downwind). The northward transect was then generated using the normalized samples A and B, H (205-m downwind), 300-m downwind average, 379-m downwind average, and M (1027-m downwind).

**Figure 10:**
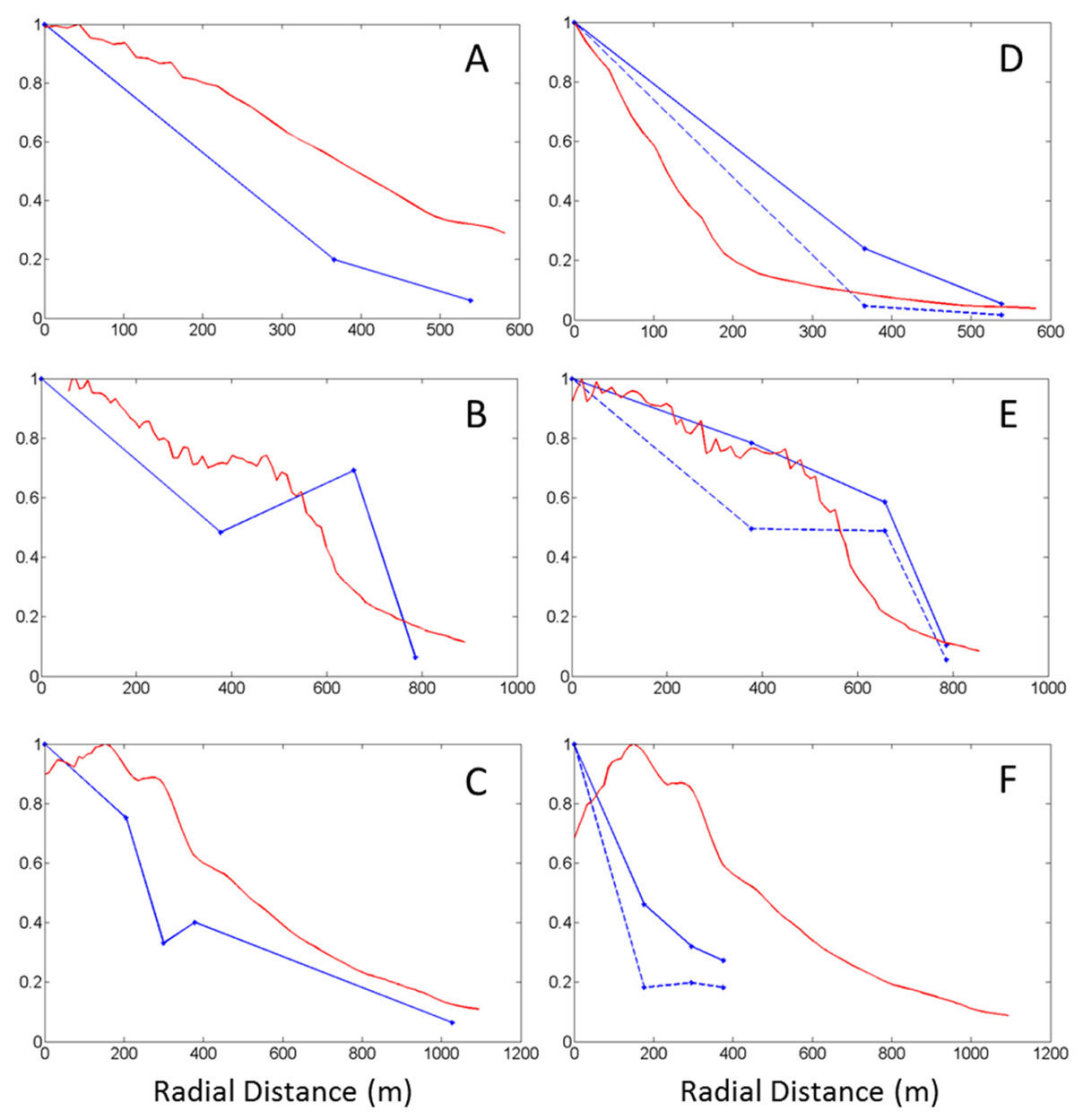
Comparison of relative decreases in arsenic (blue dashed lines) and lead (blue solid lines) mass concentrations measured by the samplers *versus* the relative decreases of deposition flux forecast by the DFM (red lines) for the southward (**A**) eastward (**B**) and northward (**C**) cross sections for the 21 April to 22 May 2014 sample period and the southward (**D**) eastward (**E**) and northward (**F**) cross sections for the 11 June to 9 July 2014 sample periods.

Results for the southward, eastward and northward cross sections for the May and June sample periods can be seen in Figure 10. The southward cross sections of the model-predicted fractional reduction in deposition follow similar trends to the observed fractional reduction of As and Pb concentrations measured in the inverted-disc samplers. For the May sampling period, the forecast overestimated the relative amount of deposition located at the sample locations E and F. This was most likely caused by strong northeasterly winds associated with a synoptic scale weather system. This weather system produced precipitation in the region, which the WRF model failed to predict. The erroneous WRF weather forecast yielded an overestimation of windblown dust transport in the southwestward direction. For June, the DFM model accurately forecasts the relative reduction in As and Pb at the E and F sample locations.

The eastward transect shows that the model-predicted fractional reduction in deposition was similar to the observed fractional reduction of As and Pb concentrations for both the May and June sampling periods for samplers C and N. However, the DFM underestimated the fractional reduction sampler D for both monthly sampling periods (Table 1). Sampler D is located just 10-m from the eastern edge of the lower tailings area and approximately 4-m lower in elevation. This proximity and lower elevation of sampler D to the lower tailings area increased the likelihood of eroded particles to gravitationally settle into this sampler. Table 1 shows that sampler D had systematically large deposition fluxes measured during both sample periods. Size distributions of the collected dust could not be determined due to the lack of total material captured by the sampler. The increased likelihood of capturing tailing material most likely skewed the fractional As and Pb deposition measured by the sampler.

The northward transect shows a similar downwind pattern of reduction in the fractional As and Pb concentration (Figure 10). The DFM overestimates the fractional reductions of Pb for the May sampling period and both As and Pb for the June sampling period. However, the DFM was significantly better estimating the relative reduction of As and Pb for the May sampling period when compared to June. For the May sampling period the DFM accurately predicted the fractional reduction in Pb concentrations for sampler M located approximately 1-km north of the tailings.

The highest concentrations of both As and Pb were measured by the inverted-disc samplers located on the tailings (A and B). In comparison, the DFM model predicts that the highest amount of tailing dust deposition should occur approximately 150 m north of sample point B. However the inverted-disc samplers show significant reduction in relative As and Pb between the tailings (A and B) and the points located about 205-m north (H and AA).

The DFM includes topographic slopes when calculating deposition rates to the surface. Using computational fluid dynamics modeling, Stovern *et al.* [10] showed that the slope of the ground significantly impacts deposition in this topographically complex region. The inverted-disc samplers were strategically placed in locations that were sloped for the northward transect. The samples located at 205-m, 300-m and 379-m were in down-sloping, up-sloping and down-sloping regions, respectively. The slope of the ground at 205-m, 300-m and 379-m is −5.7°, +15.1°, and −22.3° respectively with a slope length of 50 m. Thus, we would expect that the samples collected at 300 m should have systematically more deposition than the samplers at 379-m. However, we appear to see the opposite occurring in the May sampling period and relatively equal amounts of deposition in the June sampling period. One reason why the effects of topographic slope are not evident in the deposition patterns may be due to changes in surface roughness between the sampler locations at 300-m and 379-m. The samplers located on the up-sloping terrain at 300 m are surrounded by very sparse vegetation, usually less than a meter in height with large barren patches of soil. On the other hand, the samplers located in the down sloping region at 379-m are surrounded by significantly more vegetation including shrubs, bushes and trees that are typically 2–3 m in height. The model, however, uses a constant surface roughness of 0.1-m. Large surface roughness and obstructing objects capture airborne dust in two ways, it removes momentum from the mean flow slowing transport of airborne particulates allowing them to gravitationally settle, as well as directly capturing airborne dust through direct contact and impaction of the airborne dust. This severe change in surface roughness may explain the counterintuitive results from the samplers. Another possible reason might be the high natural variability in deposition may overwhelm the topographic effect.

#### 3.3.3. Lead Isotope Analysis

**Figure 11:**
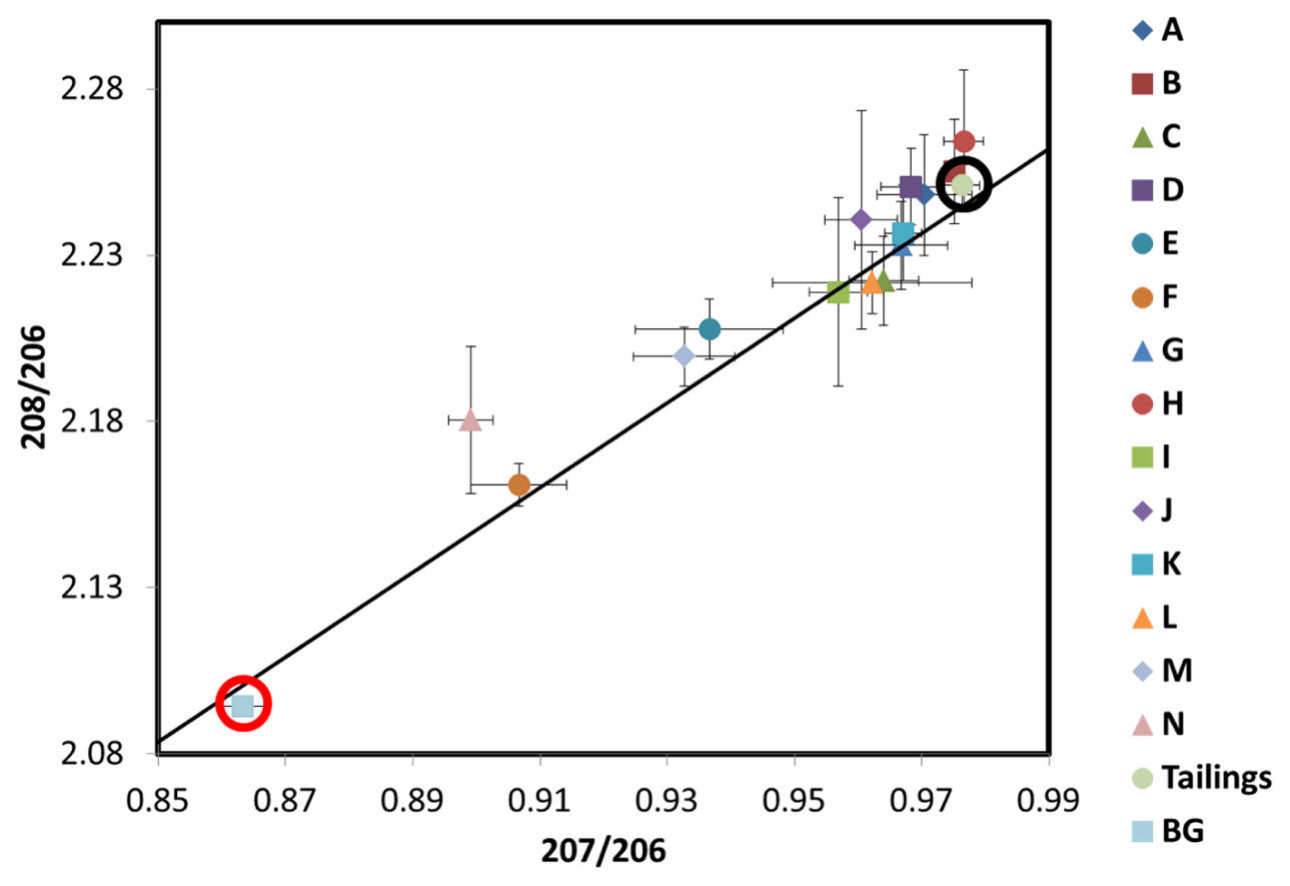
Lead isotopic ratios ^208^Pb/^206^Pb and ^207^Pb/^206^Pb for each inverted-disc sampler and a bulk sample of tailings material for the 21 April to 22 May 2014 sampling period. The letters represent the inverted-disc sample locations from Figure 1. Tailings (surrounded by a black circle), represent “fingerprint” ratios of the source. The background sample (BG, surrounded by a red circle) corresponds to topsoil (0–3 mm) collected 5 km from the source and represents the natural Pb isotopic “fingerprint” of the region. Error bars represent standard deviations from triplicate samples. Additionally included is the growth curve (solid line) adapted from Chen *et al.* [23], that represents changes in lead isotopic composition with time due to radiogenic production from isotopes of uranium and thorium, which encompasses all possible Earth samples.

**Figure 12:**
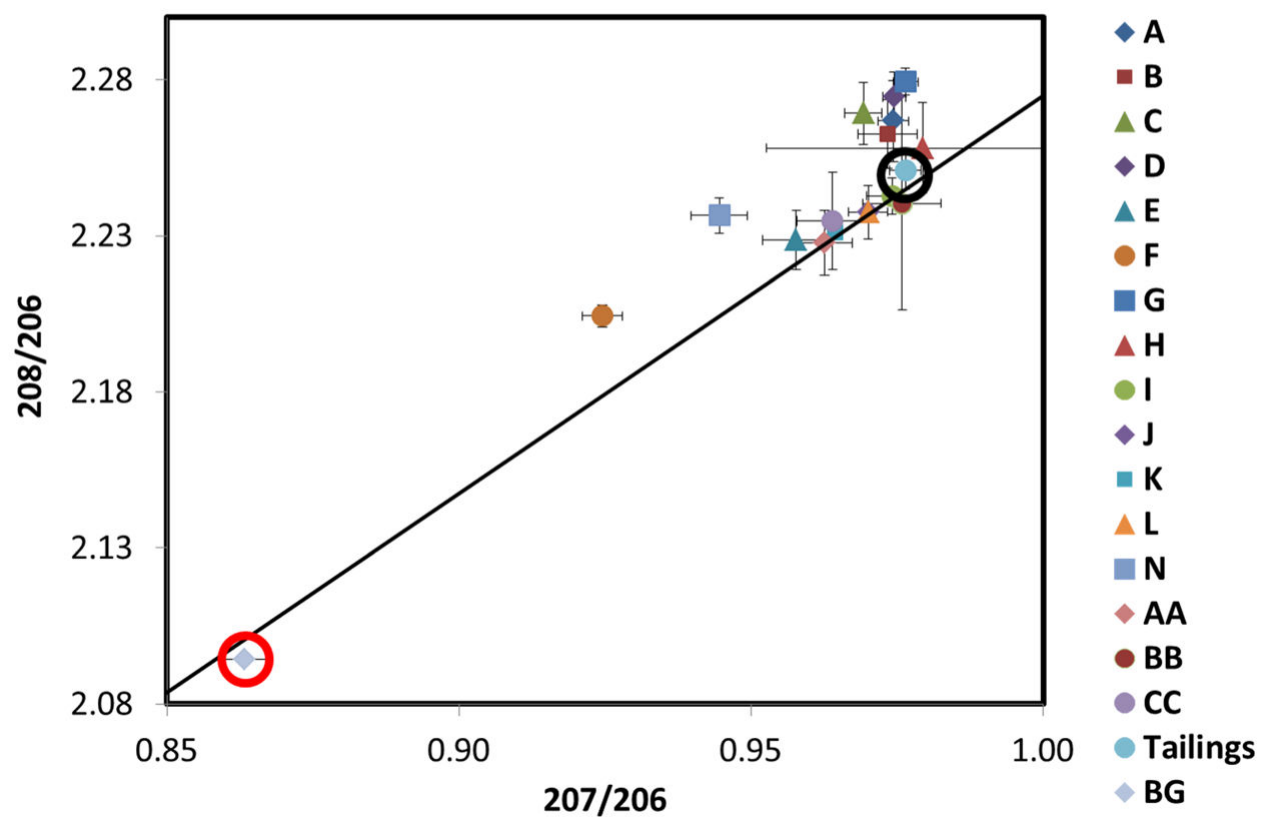
Lead isotopic ratios ^208^Pb/^206^Pb and ^207^Pb/^206^Pb for each inverted-disc sampler and a sample of tailings topsoil for the 11 June to 9 July 2014 sampling period. The letters represent the inverted-disc sample locations from Figure 1. Tailings (surrounded by a black circle), represent “fingerprint” ratios of the source. The background sample (BG, surrounded by a red circle), corresponds to topsoil (0–3 mm) collected 5 km from the source and represents the natural Pb isotopic “fingerprint” of the region. Error bars represent standard deviations from triplicate samples. Additionally included is the growth curve (solid line) adapted from Chen *et al.* [23].

Figures 11 and 12 show the lead isotopic ratios for the dust collected on the inverted-disc samplers for the May and June sampling periods, respectively. Dust samples A and B located on the tailings themselves have the same isotopic composition as the bulk tailings sample, implying the airborne lead captured in the samplers originated exclusively from the tailings, as expected.

Samples with lower isotopic ratios are the consequence of mixing between the tailings source and regional background. For the May sampling period, the samples that have the lowest isotope ratio are F and N. Sample F is the most southern sample located approximately 300-m from the southern edge of the tailings while N the eastern most sample is located approximately 150-m from the eastern-most edge of the lower tailings area. The small contribution of tailings lead measured in these samplers matches the monthly wind patterns that predominantly transported dust northward, away from the samplers. Samples M and E also have a significantly lower isotopic ratios than the tailing samples. It is interesting to note that sample E which is located only about 200 m from the southern edge of the tailings has the same fractional contribution of tailings lead as the sampler located 1 km north, as expected from the prevailing winds. Also, the source of lead captured by the inverted-disc sampler N, located along AZ highway 69 which separates the Iron King tailings and the town of Dewey-Humboldt, had a smaller tailings contribution than the sampler located 1 km north of the tailings.

For the June sampling period, the lead isotopic signatures were significantly closer to the tailings bulk sample when compared to the May sampling period. The samples with the lowest isotopic ratios included samples F, N and E. Sample F had the lowest lead contribution from the tailings, this matches the results from the May sampling period. However, more of the lead measured in sample F was sourced from the tailings compared to May. For the eastern most sampler, N and southern sampler E, the isotopic ratios were closer to the tailings signature as well, while still maintaining the lowest isotopic ratios of all the June samplers.

It is interesting to note that the June samplers had significantly higher lead concentrations and isotopic ratios when compared to May. This was caused by a precipitation event that occurred on April 27. This precipitation event significantly increased the tailings moisture content, minimizing wind erosion and reducing windblown lead deposition for the May sample period. In the June sample period the tailings had not received precipitation in over a month which significantly increasing the erosion potential, which resulted in more tailings sourced lead deposition causing the increase in lead concentrations and also shifting isotopic fingerprints closer to the tailings isotopic signature. This shows that local weather patterns including predominant wind directions and precipitation have a significant effect on the deposition of windblown dust from the Iron King tailings impoundment.

**Figure 13:**
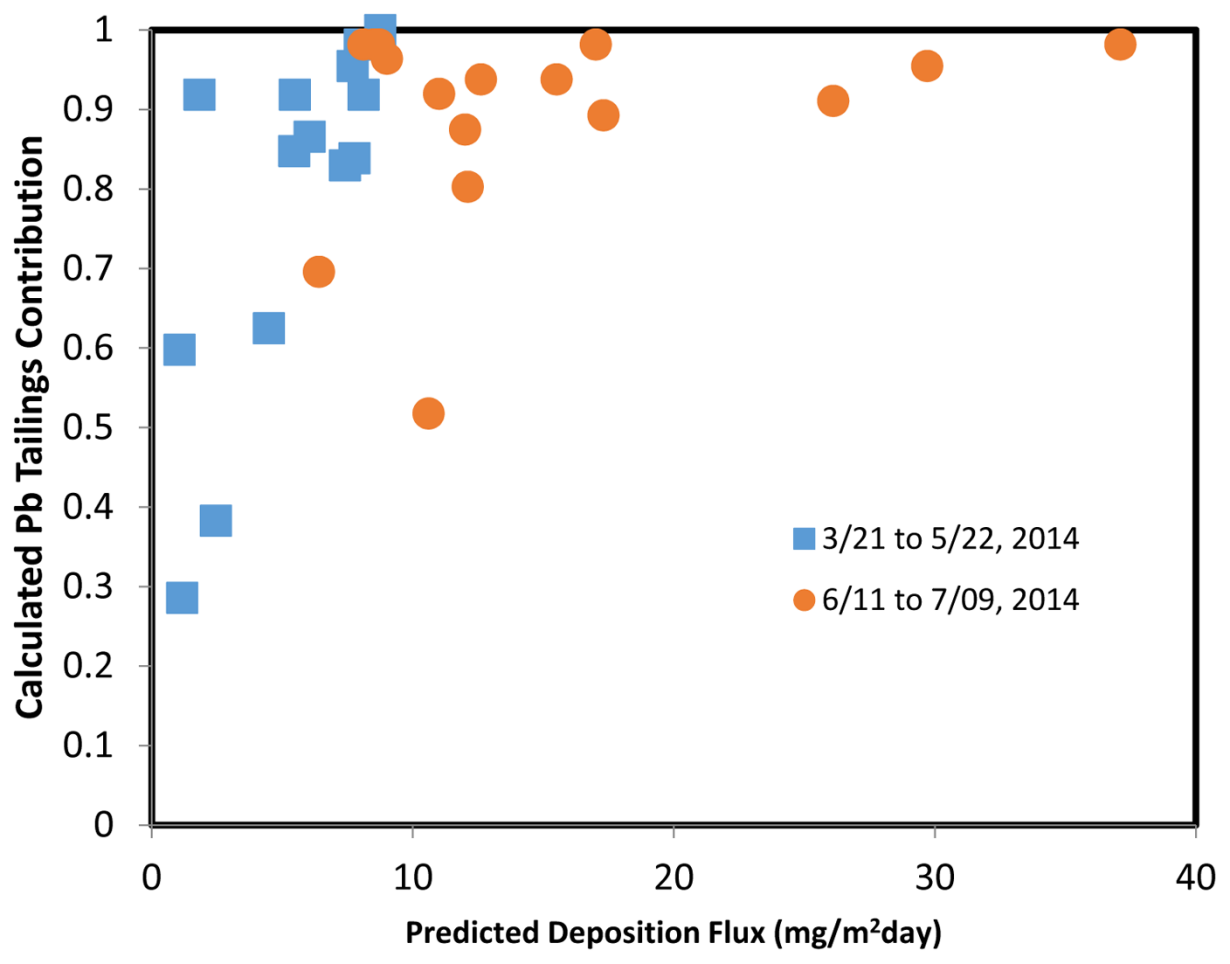
Calculated tailings contribution of deposited lead in inverted-disc samplers as a function of the model-predicted dust deposition flux for the two different sampling periods in 2014. Tailings contribution (1 for tailings, 0 for background) are calculated from the 207/206 isotopic ratios reported in Figures 11 and 12.

The lead isotope ratios can be used to calculate the contribution of deposited dust that originates in the tailings. Assuming only two different lead sources (tailings and background), the calculated fractional Pb tailings contribution was defined here as the ratio between the difference in the 207/206 ratio between the samples and the background, divided by the difference between the tailings and the background. Results are plotted as a function of predicted dust deposition fluxes in Figure 13, for the two different sampling periods. Despite the scatter, a clear increasing relation is obtained between the proportion of dust originating in the tailings and the total deposition flux of tailings materials predicted by the model.

**Figure 14:**
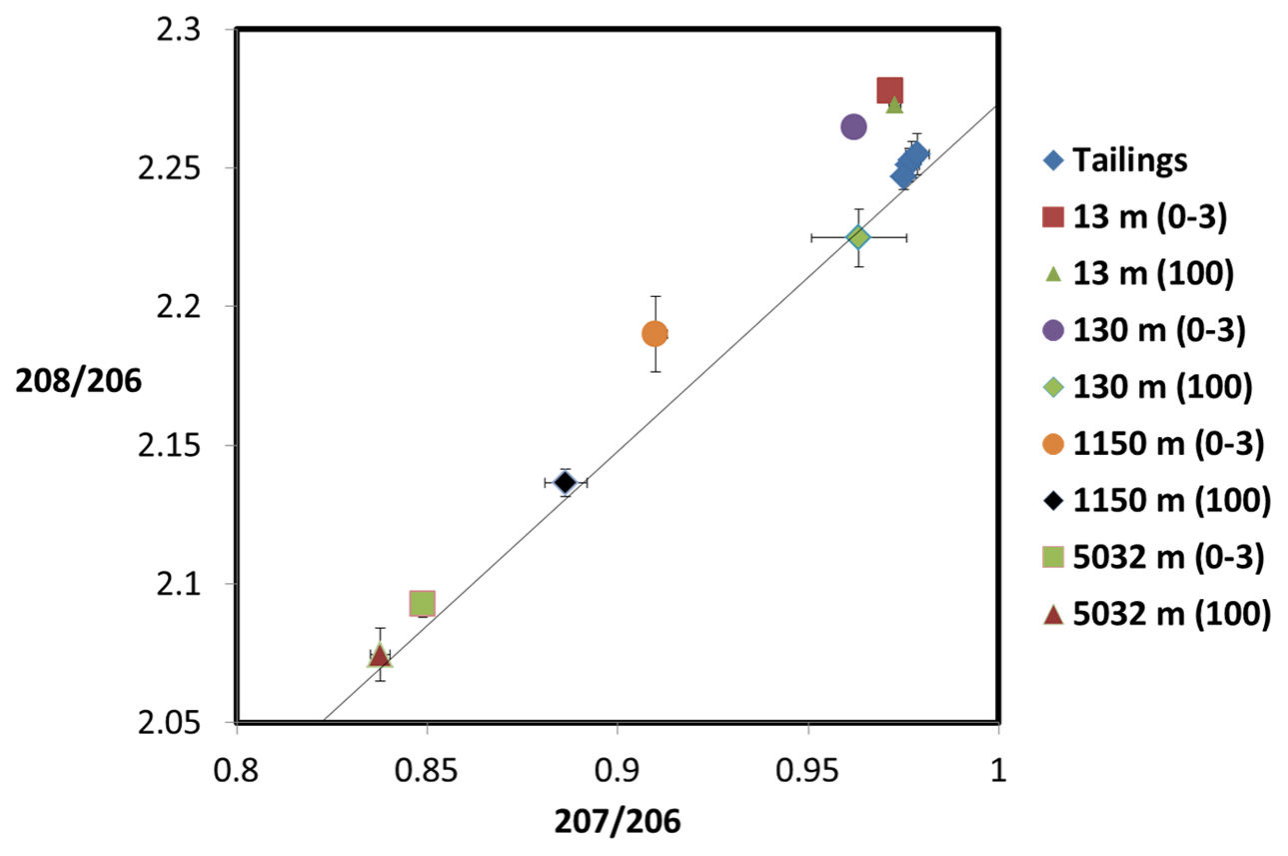
Lead isotopic ratios for soil samples at different distances from the mine tailings (indicated). Numbers in parentheses represent the depth of the sample in mm. Error bars represent standard deviations from triplicate samples. Additionally included is the growth curve (solid line) adapted from Chen *et al.* [23].

Lead isotopic ratios for the soil samples at different levels collected along the NE transect (Figure 7) are shown in Figure 14. Samples located 130 m from the tailings were collected in up-sloped terrain and their lead signatures are close to the tailings, indicating that the relatively high concentrations of lead (Figure 7) are a consequence of dust transport from the tailings. At this distance, results from the DFM model also point to a high deposition of tailings dust (Figure 10). It is interesting to point out that even at a depth of 100 mm, lead isotope analysis points to the tailings as main contributor of the metal in the soil. At longer distances from the tailing (1150 m), topsoil and 100-mm depth soil have significantly different signatures, with deep soil reflecting a higher contribution from the 5-km background. The sampling point located 13 m from the tailing has the same lead isotope ratios as the tailing topsoil, as expected, but isotopic ratios decrease monotonically with distance towards the background site located 5 km from the tailings.

## 4. Conclusions

The DFM is designed to utilize weather forecasts to predict the deposition of fugitive PM_27_ dust originating from the Iron King tailings impoundment. By comparing the DFM predicted PM_27_ deposition to arsenic and lead tracers collected by the inverted-disc samplers, it has been shown that the DFM captures trends on spatial variations of the deposition patterns in the surrounding region up to 1 km distance from the tailings. The effects of topography on deposition still need adjustment due to the complex variations of surface roughness within the region. In addition, the difficulties associated in directly quantifying PM_27_ deposition using inverted-disc samplers leaves room for additional investigation into the absolute deposition quantities provided by the DFM. However, combining the deposition patterns generated by the DFM and the known concentrations of arsenic and lead in tailing dust we can provide relative estimates of arsenic and lead deposition rates near the tailings impoundment. These estimates of deposition should improve the characterization of potential health impacts caused by windblown transport from the tailings. The methodology employed in this work can be generalized to other contaminated sites from which dust transport to surrounding communities can be assessed in terms of a potential route for human exposure. Dust emission quantification relied on weather forecasting and empirical relations for emitted dust fluxes while transport and deposition were predicted by following particle trajectories in the forecast wind field. This approach led to a model that can be used to forecast the rate of transport and deposition of contaminants from the tailings to the local environment.

## Figures and Tables

**Table 1:** Measured dust (PM_27_) mass deposition fluxes in inverted-disc samplers.

Location	4/21 to 5/22 Observed Deposition (mg/m2/day)	4/21 to 5/22 Predicted Deposition (mg/m2/day)	6/11 to 7/09 Observed Deposition (mg/m2/day)	6/11 to 7/09 Predicted Deposition (mg/m2/day)
A	15.6	7.69	17.0	4.48
B	24.2	7.96	37.1	6.11
C	6.9	6.04	26.1	4.66
D	26.0	1.82	29.7	1.36
E	9.4	4.48	12.1	0.53
F	14.2	2.45	10.6	0.27
G	12.3	8.11	8.5	8.21
H	18.2	8.75	8.1	8.67
I	10.1	7.39	12.6	7.02
J	16.2	7.76	9.0	7.54
K	30.0	5.47	17.3	5.16
L	16.3	5.45	15.5	5.15
M	16.0	1.06	N/A	N/A
N	32.5	1.16	6.4	0.68
AA	N/A	N/A	12.0	7.77
BB	N/A	N/A	8.7	7.43
CC	N/A	N/A	11.0	4.81

**Table 2:** Measured total As and Pb concentrations measured in the dust collected by the inverted-disc samplers. Locations are shown in Figure 1.

Location	4/21 to 5/22 Period Pb (ppm)	6/11 to 7/09 Period Pb (ppm)	6/11 to 7/09 Period As (ppm)
A	332	903	1826
B	981	1743	3856
C	317	1037	1406
D	454	775	1389
E	130	317	132
F	39.3	71.2	46.4
G	506	1614	1311
H	482	838	588
I	217	435	586
J	218	383	504
K	234	342	465
L	292	439	649
M	41.9	N/A	N/A
N	41.2	138	156
AA	N/A	384	449
BB	N/A	453	595
CC	N/A	302	435
